# Congenital eye malformations associated with extensive periocular neural crest apoptosis after influenza B virus infection during early embryogenesis

**Published:** 2009-12-19

**Authors:** Bo-Yie Chen, Han-Hsin Chang, Shyan-Tang Chen, Zih-Jay Tsao, Shang-Min Yeh, Chia-Yung Wu, David Pei-Cheng Lin

**Affiliations:** 1 School of Optometry, Chung Shan Medical University, Taichung, Taiwan; 2 School of Nutrition, Chung Shan Medical University, Taichung, Taiwan; 3 School of Medical Laboratory and Biotechnology, Chung Shan Medical University, Taichung, Taiwan; 4 Department of Ophthalmology, Chung Shan Medical University Hospital, Taichung, Taiwan

## Abstract

**Purpose:**

Congenital eye malformations are a leading cause of blindness in children. Influenza virus infections prevail worldwide and have been implicated in congenital defects. Infections acquired during gestation may disrupt eye morphogenesis. We investigated the effects of influenza B virus infection on eye malformations during early embryogenesis.

**Methods:**

Chick embryos were exposed to influenza B virus at Hamburger-Hamilton stage 9. Maternal infection was conducted by exposing pregnant ICR mice to influenza B virus at the embryonic gestation stage E 5.0. After infection, virus RNA distribution was detected by in situ hybridization at various developmental stages. The distribution of periocular neural crest cells and the extent of apoptosis were examined by immunohistochemical staining, in correlation with eye malformations.

**Results:**

Microphthalmos and anophthalmos, together with neural tube defects, were found in the chick and mouse embryos following the infections. The viral RNA was detected in the head neuroepithelium, optic vesicle, periocular mesenchyme, and the forming ventricles of the developing brain. Immunohistochemical staining revealed aberrant neural crest distribution and extensive apoptosis in the head surface ectoderm, periocular mesenchyme, and optic vesicle in the chick embryos. Furthermore, transplacental infection was confirmed by the presence of viral RNA in the mouse fetuses, with eye and neural tube defects similar to those found in the chick embryos after experimental infections.

**Conclusions:**

Influenza B virus may act as a teratogen to cause aberrant periocular neural crest cell contribution to eye development and extensive apoptosis, resulting in congenital eye malformations.

## Introduction

Severe forms of ocular malformation, such as anophthalmia (absence of the eye), microphthalmia (very small eye), and coloboma (failure to close the optic fissure), appear at a frequency of approximately 30 per 100,000 people in the human populations [1-3] or 70 incidents per 480,000 live births in the United States [4]. The pathogenesis of these congenital ocular malformations is clearly multifactorial and may involve genes inactivation or mutations, such as those found in *SOX2*, *PAX6*, *CHX10*, and *OTX2*, leading to disruption of eye morphogenesis during embryonic development [1,3]. Aside from genetic defects, infections acquired during gestation may also lead to eye malformations. In some earlier reports, fetal anophthalmos and microphthalmos had been correlated with maternal influenza infections, along with defects in the neural tube [5,6] and central nervous system [7,8]. In a more recent study, Czeizel et al. (2008) [9] reported that in 1,349 cases of multiple congenital abnormalities without recognizable genetic and teratogenic syndromes, 181 had a possible association with influenza, common cold, or other secondary complications. The data indicated a positive correlation between severe anophthalmos or microphthalmos prevalence and maternal influenza infections [8], despite that no direct cause-to-effect evidence was given.

In a previous study, we had shown that influenza B virus (B/Taiwan/25/99) infection could lead to teratogenesis in early chick embryos [10]. By injecting the virus into sub-blastodermal space of chick embryo at Hamburger-Hamilton stage 9 [11], gross malformations in the eye and the brain were observed at 48 h after the procedure, in contrast to the normal development of the sham-infected controls. We further confirmed that influenza B virus targeted directly at the chick embryos by showing the distribution of viral RNA in the neural retina, brain, head surface ectoderm, spinal cord, and lung bud [10]. Despite previous findings, the cellular mechanisms underlying the congenital ocular abnormalities in this influenza virus–infected chick embryo model are still poorly understood. Besides, whether influenza virus may cause transplacental infection and whether the teratogenic effects observed in the chick embryos may reflect the status of mammalian embryos after infection remain to be elucidated.

In the present study, we further characterized the teratogenic effects of influenza B virus infection using both the chick and mouse models. We found anophthalmos or microphthalmos after the experimental infections and the viral RNA distribution was correlated with eye malformations. Furthermore, transplacental infection was confirmed in the mouse embryos, with similar eye malformations comparable to those found in the chick embryos. Our data indicated that influenza virus infection is teratogenic during eye morphogenesis.

## Methods

### Preparation of influenza B virus

The influenza virus B/Taiwan/25/99 strain was isolated and its identity confirmed by nucleic acid sequencing as previously described [10]. The virus was prepared in Madin-Darby canine kidney (MDCK) cells and tittered by plaque assay on MDCK cells, followed by dilution of the virus in EMEM to a concentration of 5×10^8^ plaque-forming units (pfu)/ml and stored in a –70 °C freezer. The preparations were thawed shortly before use. No re-frozen virus was used in this study.

### Obtainment and infection of chick and mouse embryos

Fertilized pathogen-free chicken eggs were purchased from Jin-Dan Incubation Services, Taichung County, Taiwan and incubated at 38.5 °C in order for the embryos to develop. At Hamburger-Hamilton stage 9, an aliquot of 20 μl of the prepared influenza B virus was carefully injected in ovo with a 30-gauge needle into the sub-blastodermal space without damage to the embryo. Mock injections of 20 μl EMEM without virus were performed as sham-infected controls. The infected and non-infected chick embryos were returned to separate incubators at 38.5 °C for further incubation until analysis.

The mouse embryos were obtained by mating ICR mice purchased from National Laboratory Animal Center, Taipei, Taiwan. For mouse embryo infections, an aliquot of 200 μl of the prepared virus was injected into the tails of pregnant ICR mice at E 5.0 (noon on the day of finding a vaginal plug was designated as E 0.5). Mock infections with the same amount of EMEM injection were performed as the controls, but without the virus. After injection, each pregnant mouse was kept in an isolated room until further analyses.

### Cloning of influenza B viral hemagglutinin A (HA) segment

Viral total RNA was extracted, amplified by RT–PCR, and then cloned into the pCR®II-TOPO vector (Invitrogen, Carlsbad, CA) following the manufacturer’s instructions, as previously described [10]. The DIG-labeled RNA probe was synthesized for direct in situ localization of influenza B viral RNA in wholemount embryos and tissue sections after infection [10].

### In situ hybridization, immunodetection, and in situ apoptosis assay

In situ hybridization and immunodetection for embryonic tissue sections and wholemount embryos were performed according to previously published procedures [10,12,13]. The RNA probe was synthesized according to manufacturer's instructions (Roche Applied Science, Indianapolis, IN). The probe specificity for influenza B hemagglutinin (HA) sequence was examined by blast analyses on NCBI (National Center for Biotechnology Information) database with left primer (BHA^+^: 5′-CGA ATC TGC ACT GGG ATA ACA TC-3′) or right primer (BHA^-^: 5′-TGC ACC ATG TAA TCA ACA ACA A-3′) or with both primers for the cDNA cloning. The blast results showed specificity only to the HA sequence of influenza B virus, indicating that cross-hybridization with influenza A viral genome is least likely. The sham-infected control embryos were processed simultaneously with the influenza B-infected embryos following the same hybridization procedures. For immunodetection, the antibodies were diluted as: anti-DIG, 1/3500 (Roche Applied Science, Indianapolis, IN); anti-HNK-1, 1/250 (Sigma-Aldrich, St Louis, MO). In situ apoptosis assay (TUNEL assay) was processed following the manufacturer’s instruction (Millipore, Billerica, MA). For both immunodetection and TUNEL assay, the sham-infected control embryos were processed in parallel with the infected embryos following the same procedures. Some infected embryos and sham-infected control embryos were processed without antibody to serve as negative controls for the HNK-1 immunodetection, or without adding terminal deoxynucleotidyl transferase to serve as negative controls for the TUNEL assay, or without adding RNA probe to serve as negative controls for the in situ hybridization. All positive results were performed at least twice for confirmation.

## Results

### Ocular malformations after infections are consistent with influenza B viral RNA distribution

**Figure 1:**
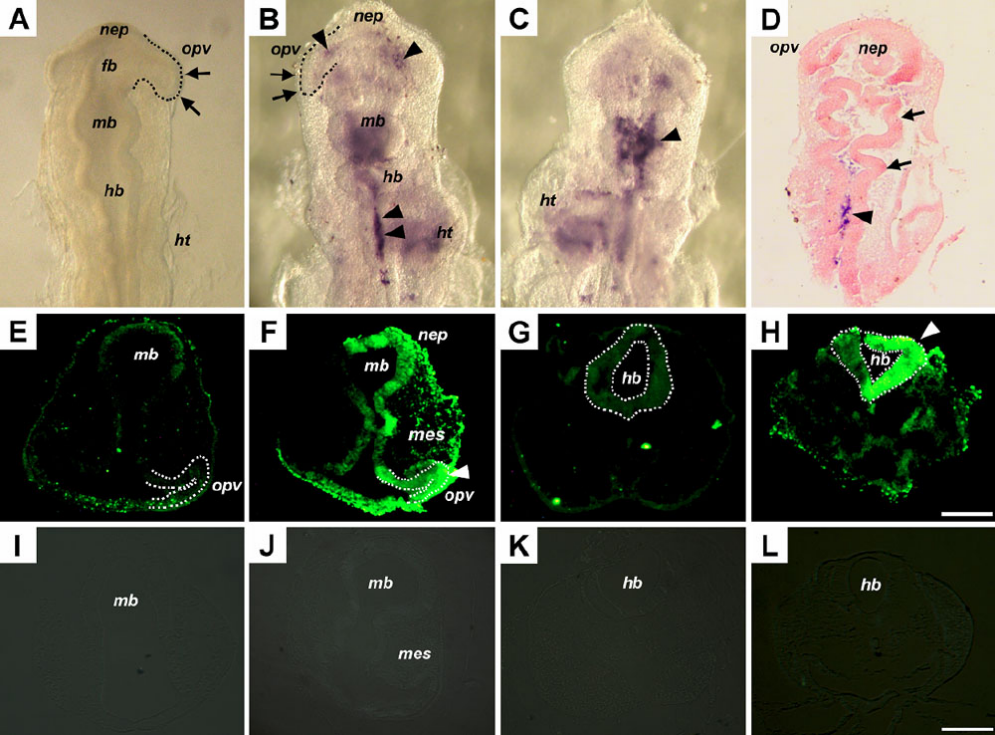
Eye malformation and neural tube defect after influenza B virus infection in the chick embryo model. **A**, **E**, **G**, **I**, and **K** were from sham-infected control embryos. **B**, **C**, and **D** were from an infected embryo. **F**, **H**, **J**, and **L** were from another infected embryo. **A**-**D** were processed through in situ hybridization with a RNA probe containing influenza B virus hemagglutinin segment. The presence of viral RNA was indicated by blue or purple signals. **E**-**H** were processed through in situ apoptosis assay on transverse tissue sections, with apoptotic cells indicated by green fluorescence signals. **J**-**L** were also processed through in situ apoptosis assay on transverse tissue sections, but without adding terminal deoxynucleotidyl transferase. **A**: A sham-infected control embryo. **B**: Dorsal view of an infected embryo exhibited abnormal optic vesicle development (indicated by arrows and dotted line), as compared to the control embryo showing normal optic vesicle development in **A** (indicated by arrows and dotted line). The viral RNA was detected in the head neuroepithelium, optic vesicle, periocular mesenchyme, and in the heart. **C**: Ventral view of the same embryo in **B**, showing evident presence of viral RNA in the head neuroepithelium. **D**: Coronal section of the embryo in **B**, showing the presence of viral RNA in the neural tube (indicated by arrowhead) and the forming ventricles of the developing brain enclosed by twisted neuroepithelium (indicated by arrows). **E** and **G**: Midbrain and hindbrain sections of a sham-infected control embryo showing a few apoptotic cells normally observed during early chick embryogenesis. **F** and **H**: Extensive apoptotic cells in the midbrain and hindbrain sections of a virus-infected embryo. **I** and **K**: Negative controls for TUNEL assay on sham-infected embryos corresponding to **E** and **G** and showing no background signals. **J** and **L**: Negative controls for TUNEL assay on virus-infected embryos corresponding to **F** and **H** and showing no background signals. Abbreviations: fb, forebrain; hb, hindbrain; ht, heart; mb, midbrain; mes, mesenchyme; nep, neuroepithelium; opv, optic vesicle. Scale bar: 50 µm.

**Figure 2:**
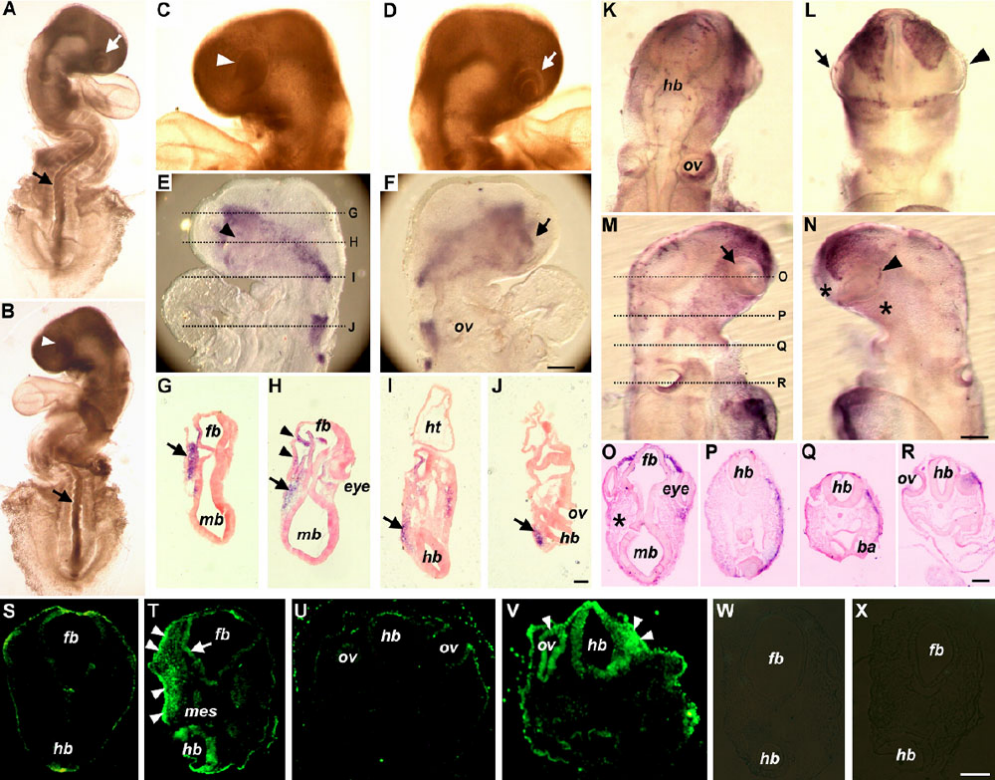
Teratogenic effects of influenza B virus infection in the chick embryo model. The images in **A**-**J** are from the same embryo, and those in **K**-**R** are from another embryo. **A**-**J**: A virus-infected embryo at Hamburger-Hamilton stage 15 showing gross malformations (**A**-**D**), localization of viral RNA on whole mount preparation (**E** and **F**) and on tissue sections (**G**-**J**). **K**-**R**: Distribution of HNK-1 positive neural crest cells in a virus-infected embryo at Hamburger-Hamilton stage 13 showing neural crest cell population in the dorsal view (**K**), ventral view (**L**), lateral views (**M** and **N**), and in tissue sections (**O**-**R**). **S** and **U**: Forebrain and hindbrain sections of a sham-infected control embryo at Hamburger-Hamilton stage 15 showing few apoptotic cells. **T** and **V**: Extensive and unilateral distribution of apoptotic cells in the forebrain and hindbrain sections of a virus-infected embryo at Hamburger-Hamilton stage 15. **W**: A negative control for TUNEL assay on sham-infected embryo corresponding to **S** and showing no background signals. **X**: A negative control for TUNEL assay on virus-infected embryo corresponding to **T** and showing no background signals. **A** and **B**: Lateral view of asymmetric eye development (indicated by white arrowhead and white arrow) and twisted neural tube in the same embryo. **C** and **D**: Magnified head region showing asymmetric eye development. **E** (on focus) and **F** (out of focus): Asymmetric eye development and unilateral distribution of viral RNA as shown by the blue signals on the same focus plane. **G**-**J**: Areas of transverse sections as indicated in **E**, showing evident unilateral distribution of viral RNA in the head mesenchyme (indicated by arrow). Note that viral RNA was concentrated on the side of eye malformation (indicated by arrowheads in **H**). **K**-**N**: Asymmetric distribution of neural crest cells in a virus-infected embryo. The normal eye (arrow-indicated) was surrounded by more neural crest cells than the malformed eye (arrowhead indicated). **O**-**R**: more evident asymmetric neural crest cell distribution, as shown in blue signals, was seen on sections of the areas indicated in **M**. The asterisk in **O** indicates unilateral shortage of neural crest cells as compared to the other side. **T** and **V**: More apoptotic cells were detected in the surface ectoderm, neuroepithelium, and mesenchyme as compared to the sham-infected controls (**S** and **U**). Abbreviations: ba, branchial arch; fb, forebrain; hb, hindbrain; ht, heart; mb, midbrain; mes, mesenchyme; ov, otic vesicle. Scale bars: **F** and **N**, 200 μm; **J** and **R**: 100 μm; **V** and X, 50 μm.

At 48 h after infection, 21 out of 25 influenza B virus-infected chick embryos exhibited eye, brain, and neural tube malformations (Figure 1A-D; also Figure 2A-D). To elucidate how influenza B virus affects chick embryo development, we located the distribution of viral RNA by using in situ hybridization with an RNA probe specific for the HA segment of influenza B virus as previously described [10]. The viral RNA was detected in the head neuroepithelium, optic vesicle, periocular mesenchyme, and in the forming ventricles of the developing brain at 48 h after infection (Figure 1B-D). At 96 h after infection (Figure 2E-J), unilateral distribution of the viral RNA was commonly seen in the head region of the influenza B virus-infected chick embryos. The unilateral distribution of viral RNA appeared to be due to single-sided exposure of the embryos to the virus, as chick embryos normally turn during development, resulting in unilateral contact with the virus that had been injected into the sub-blastodermal space. This unilateral effect within the same embryo allowed for comparison between the side facing directly to the virus and the other side without direct virus exposure. With this regard, we tried to find unilateral teratogenic effects in the virus-infected embryos. The results showed that a high proportion (14/25) of influenza B virus-infected chick embryos exhibited eye malformations on a single side (Figure 2C-D,M-N). The eye malformations, when compared with the normal eye development in the sham-infected controls, are characteristic of being at earlier developmental stages and of smaller eye sizes that are morphologically similar to microphthalmos. As represented in Figure 2C, it was evident that the eye (indicated by arrowhead) was not developed to the same stage as that seen in Figure 2D where the lens placode (indicated by arrow) was apparently discernible, even though both eyes were from the same embryo. Similarly, the eye development in Figure 2N (indicated by arrowhead) had not reached to the same developmental stage as compared to that in Figure 2M (indicated by arrow). Such asymmetric eye malformations matched well with the unilateral distribution of viral RNA in the influenza B virus-infected chick embryos. As detected in Figure 2E-F on the same focus plane, the viral RNA shown by blue signals was more evident on one side of the infected embryo (Figure 2E; the side on the focus plane) than on the other side (Figure 2F; the side not on the focus plane). This asymmetric viral RNA distribution was seen more evidently on tissue sections (Figure 2G-J; viral RNA signals indicated by an arrow). Interestingly, we also found that eye malformations were consistently correlated with the presence of viral RNA in the periocular mesenchyme (arrow-indicated in Figure 2G, H), as represented by one side of the embryo without evident eye formation in Figure 2H (indicated by arrowheads), in contrast to the other side where a normal eye was developed.

### Influenza virus infection induces extensive apoptosis in optic vesicle and periocular mesenchyme

Normal eye development involves growth of the surface ectoderm, neuroepithelium, and periocular mesenchyme and the interactions among these three components. Since the influenza B virus-infected chick embryos exhibited relatively smaller and primitive eye structures and the morphogenesis of the optic vesicle and lens placode was markedly disrupted or delayed, it is tempting to elucidate which component of the developing eye is primarily affected by the infection and how the mutual interactions among the three eye components are affected. To address this question, we performed in situ apoptosis assay and found that extensive apoptotic cells were unilaterally distributed on transverse tissue sections in the head region of the influenza B virus-infected chick embryos, as represented by embryos at Hamburger-Hamilton stage 10 (Figure 1E-H) and stage 15 (Figure 2S-V). Extensive apoptosis was found, not only in the neuroepithelium of the optic vesicle (Figure 1F, indicated by arrowhead) and surface ectoderm (Figure 2T,V; indicated by arrowhead), but also in the head and periocular mesenchyme (Figure 1F and Figure 2T; indicated by mes). No comparable apoptosis was found in the sham-infected embryos (Figure 1E,G and Figure 2S,U) that showed only minimal signals pertaining to naturally occurred apoptotic activities during normal chick embryogenesis. Parallel TUNEL assay procedures were performed without adding terminal deoxynucleotidyl transferase on the influenza B virus-infected chick embryos (Figure 1J,L and Figure 2X) and on the sham-infected embryos (Figure 1I,K and Figure 2W), with no comparable signals found in these controls.

### Influenza virus infection causes aberrant periocular neural crest cell distribution

During normal eye development, a substantial population of head neural crest cells migrates and contributes to the periocular mesenchyme. Abnormal migration and differentiation of these neural crest cells have been implicated in eye dysgenesis [14,15]. We have observed insufficient lens placode formation and extensive apoptosis in the periocular mesenchyme in the chick embryos after influenza B virus infection and both were found unilaterally. The insufficiency of lens formation might be resulted from aberrant distribution of neural crest cells, leading to abnormal ocular development eventually. To further elucidate this hypothesis, we performed immunodetection of HNK-1, an early molecular marker for the chick neural crest cells, in the influenza B virus-infected chick embryos. We found that HNK-1 signals were asymmetrically distributed in most of the influenza B virus-infected chick embryo heads, as represented by Figure 2K-R. Viewed ventrally in the infected embryo in Figure 2L, more HNK-1 positive neural crest cells were detected, as indicated by blue signals, on one side where the lens placode was formed normally (indicated by arrow), as compared to the other side with less evident lens placode formation (indicated by arrowhead). The shortage of periocular neural crest cells was also detected in the lateral views of the same infected embryo (Figure 2M,N). More neural crest cells were found surrounding the normal eye on one side of the embryo (Figure 2M; indicated by arrow) than those (indicated by asterisks) surrounding the abnormal eye (Figure 2N; indicated by arrowhead) on the other side of the same embryo. The differential distribution was seen most evidently in tissue sections (Figure 2O-R). Particularly, in Figure 2O, one side of the embryo contained less periocular neural crest cells (indicated by asterisk) than the other. Our findings support the hypothesis that unilateral insufficiency of neural crest cells in the influenza B virus-infected chick embryos leads to unilateral eye malformations.

### Transplacental infection can be achieved by influenza B virus

**Figure 3:**
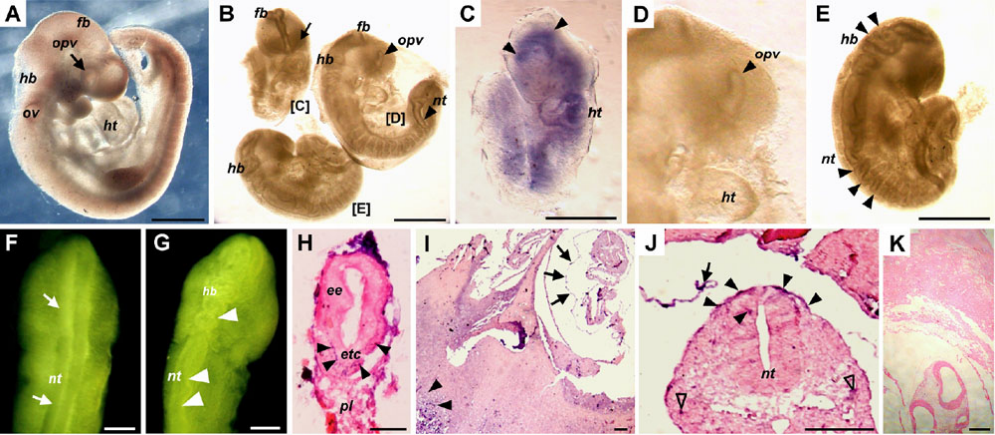
Teratogenic effects of influenza B virus infection in the mouse embryo model. **A** and **F** are from sham-infected control embryos. **B**-**E** and **G**-**K** are from virus-infected embryos. **A**-**G** are wholemount embryo preparations. **J**-**K** are embryo tissue sections. **A**: A mouse embryo showing normal development at E9.5 after sham infection at E5.0. **B**: Reduction of embryo size in 3 virus-infected embryos as compared to the sham-infected control in **A** at the same gestation stage. **C**: The blue signals indicate the localization of viral RNA in neuroepithelium and as indicated by arrowheads, in an infected mouse embryo. **D**: An example of abnormal optic vesicle (indicated with arrowhead), as compared to the normal optic vesicle in **A** (indicated with arrow) at the same gestation stage. **E** and **G**: Twisted head neuroepithelium and trunk neural tube (indicated by arrowheads) as compared to the normal neural tube in **F**. **H**: Viral RNA distribution in embryonic endoderm (indicated by arrowheads). **I**: Viral RNA distribution in the amniotic membrane (indicated by arrows) and in placenta (indicated by arrowheads). **J**: Viral RNA distribution in trunk surface ectoderm (indicated by arrowheads) and in the neural tube (indicated by an arrowhead). **K**: A negative control for the in situ hybridization procedures showing no background signals. Gestation stages of embryos: **A**-**G**, E9.5; **H**, E6.0; **I** and **J**, E6.5. Abbreviations: ba, branchial arch; ee, embryonic endoderm; etc, ectoplacental cone; fb, forebrain; hb, hindbrain; ht, heart; nt, neural tube; mes, mesenchyme; opv, optic vesicle; ov, otic vesicle; pl, placenta. Scale bars: **A**, **B**, **C**, and **E**, 500 μm; **F** and **G**, 50 μm; **H**-**K**, 100 μm.

Despite that various eye defects were found in the chick embryo model after infection, our data would not be of clinical significance if the virus can not trespass the placenta barrier. Therefore, we examined whether transplacental infection may occur during early pregnancy in the mouse model and tried to determine whether mouse developmental defects caused by influenza B virus infection are comparable to those found in the chick embryos. We found that fetal size was commonly reduced in the mouse embryos at E9.5 after maternal influenza B virus infection at E5.0 (Figure 3A,B). Some mouse embryos (15/172) did not develop an optic vesicle after maternal influenza B virus infection at E5.0 (Figure 3B; arrow-indicated in embryo [C], as compared to its littermate in embryo [D]), representing a status similar to anophthalmos. We also found twisted neuroepithelium in the optic vesicle (71/172), the brain (81/172), and the neural tube (77/172) in the fetuses derived from maternally infected mice (Figure 3D,E,G; indicated by arrowheads). These mouse embryos generally displayed a status of microphthalmos in morphology (Figure 3B; arrowhead-indicated in embryo [D]), as compared to the controls (arrow-indicated in Figure 3A). Between E9.0 to E9.5, as shown in blue signals, the viral RNA could be detected in the fetus, notably in the diencephalic neuroepithelium (indicated by arrowheads) and the heart tissue (ht) and in the other regions (Figure 3C). In addition, we found that the viral RNA could be detected as early as E6.5 in the embryonic endoderm (Figure 3H; arrowheads), the placenta (Figure 3I; arrowheads), and the amniotic membrane (indicated by arrows in Figure 3I; also by the arrow in Figure 3J), as well as in the embryonic surface ectoderm (Figure 3J; indicated by arrowheads), neural tube (Figure 3J; arrowhead), and mesenchyme (indicated by hollow arrowheads in Figure 3J). No comparable signals were detected in the sham-infected embryos that had been processed in parallel to the embryos after maternal influenza B virus infection (Figure 3K). The results demonstrated that influenza B virus can target directly at embryos across placenta and cause eye malformations similar to those found in the chick embryos.

## Discussion

In the present study, our results showed that direct exposure to influenza B virus (B/Taiwan/25/99) can cause developmental defects in the eye, brain, and neural tube. The HA segment of the viral RNA was located in the infected embryos, indicating that influenza B virus can target directly at the embryonic tissues. The fact that we can detect influenza B virus RNA in the head surface ectoderm, diencephalic neuroepithelium, neural tube, and head mesenchyme is contrary to the notion that influenza viruses target specifically at the respiratory tract epithelium in adult mammals, including humans. However, there have been some reports demonstrating that human tissues other than respiratory tract epithelium can be targeted by influenza viruses. Nakai et al. [16] investigated glial reaction and apoptosis in postmortem brains and found increased apoptosis in neurons and glial cells in four brains with influenza encephalopathy. Levine et al. [17] showed that human Schwann cells can be infected with human influenza A virus under in vitro conditions. Another study by Brask et al. [18] demonstrated the changes in calcium currents and GABAergic spontaneous activity in cultured rat hippocampal neurons after a neurotropic influenza A virus infection. Taken together, these evidences from animal embryo models, human fetuses, and in vitro studies support influenza viruses as a teratogenic agent during pregnancy.

Eye malformations are of particular significance among the teratogenic effects caused by influenza virus infection, since they are not fatal to embryos and many lines of evidences have correlated fetal anophthalmos and microphthalmos with maternal influenza infections [5-9]. Although epidemiological surveys have already implicated influenza viruses as a teratogenic pathogen during eye morphogenesis [8], there has been no direct cause-to-effect evidence to support this hypothesis. In this study, we demonstrated that eye malformations after influenza virus infection are consistent with the distribution of viral RNA, providing a cause-to-effect evidence to correlate influenza infections with fetal anophthalmos and microphthalmos. Furthermore, we showed extensive apoptosis in the optic vesicle and periocular mesenchyme as well as aberrant distribution of neural crest cells, which may lead to anophthalmos and microphthalmos in the influenza virus-infected embryos. During embryonic development, the eye is derived from three different tissues, the anterior neuroectoderm, the neuroepithelium, and the periocular mesenchyme. The surface ectoderm later forms the lens and corneal epithelium, while the neuroepithelium gives rise to the neural retina and the pigment retinal epithelium. The third cellular population of the developing eye, the periocular mesenchyme, originates from both the head mesoderm and the cranial neural crest [19-22]. The cranial neural crest cells migrate from the dorsal head neuroepithelium and contribute critically to the developing intra- and extra-ocular structures, such as the corneal endothelium, stromal cells, iris, ciliary body, choroid, sclera, and a part of the extra-ocular muscles [19-22]. Abnormal neural crest cell differentiation and distribution has been well documented to be responsible for ocular anomalies in clinical patients and experimental animals, such as uveal coloboma [23-25], cyclopia [26], anophthalmos [27], and microphthalmos [23,24,28]. Therefore, the direct targeting by influenza B virus on head mesenchyme, particularly on periocular mesenchyme, is likely to result in extensive apoptosis, and hence the deficiency of neural crest cells, leading to the formation of anophthalmos or microphthalmos. Alternatively, the aberrant distribution of HNK-1 positive neural crest cells may reflect insufficient migration or failure of differentiation following influenza B virus infection.

Deficiency of lens placode formation may also contribute to the teratogenic effects of influenza B virus on head mesenchyme. It is known that TGF-β signaling provides significant instructive information to regulate neural crest cell migration and differentiation [29] and the lens acts as a TGF-β signaling center to control the development of eye structures derived from neural crest cells [30]. Since our data have shown that lens formation is affected in the presence of influenza B viral RNA, it is likely that the regulatory roles of TGF-β signaling on neural crest cells are diminished due to insufficiency of lens formation, leading to abnormal ocular development.

The severity of teratogenesis caused by influenza virus infection depends on the developmental stage when the infection occurs and on whether the teratogenic effects are caused directly by viral targeting or indirectly from maternal immune responses. We showed that influenza B viral RNA can be detected in the early mouse embryos, indicating that transplacental infection could happen shortly after implantation. Our results support a previous data reported by Aronsson et al. [31] who found viral RNA in the brain of offspring after maternal infection with influenza A/WSN/33 during pregnancy. Furthermore, our data in the mouse model is similar to the findings in the chick embryos, consistent with the hypothesis that influenza virus infections can lead to anophthalmos or microphthalmos.

Obviously, the underlying mechanisms involved in the teratogenic effects of influenza virus remain to be further elucidated. We do not know exactly how apoptosis in periocular mesenchyme may cause malformations in the eye and how influenza infection may alter gene expression in different areas of the developing eye. Besides, the effect of direct virus targeting has to be distinguished from indirect effects from maternal immune responses. Despite the lack of detailed information, our results demonstrate that influenza virus infection during early embryogenesis can cause eye malformations, which should serve as an alert to clinicians.
